# A Phenomenological Study on the Positive and Negative Experiences of Chinese International University Students From Hong Kong Studying in the U.K. and U.S. in the Early Stage of the COVID-19 Pandemic

**DOI:** 10.3389/fpsyt.2021.738474

**Published:** 2021-12-13

**Authors:** Agnes Yuen-kwan Lai, Shirley Man-man Sit, Stanley Kam-ki Lam, Asa Ching-man Choi, Denise Yee-shan Yiu, Theresa Tze-kwan Lai, Mary Sau-man Ip, Tai-hing Lam

**Affiliations:** ^1^School of Nursing, The University of Hong Kong, Hong Kong, Hong Kong SAR, China; ^2^School of Public Health, The University of Hong Kong, Hong Kong, Hong Kong SAR, China; ^3^School of Health Sciences, Caritas Institute of Higher Education, Hong Kong, Hong Kong SAR, China; ^4^Department of Medicine, The University of Hong Kong, Hong Kong, Hong Kong SAR, China

**Keywords:** stress, coping, university students, international, positive and negative experience, phenomenological study, COVID-19, Chinese

## Abstract

**Background:** The COVID-19 pandemic has caused distress in students globally. The mental health of international students studying abroad has been neglected during the COVID-19 pandemic, especially Chinese students who have been unfairly targeted.

**Objective:** To explore and document the positive and negative experiences of a group of Hong Kong Chinese international students studying in the U.K. and U.S. from an insider perspective in the early stage of the COVID-19 pandemic.

**Methods:** The qualitative study used four 1.5-h online focus group interviews of 20 Chinese international students from Hong Kong aged 18 or older studying in universities in the United Kingdom or the United States, from 3 May to 12 May 2020. A framework approach with a semi-structured interview guide was used to reflect students' stressors, cognitive appraisals, coping, and outcomes (negative impacts and positive gains), in the early stages of COVID-19. Different strategies were used to ensure the credibility, dependability, confirmability, and transferability of the study. Transcripts were analyzed using qualitative thematic content analysis.

**Results:** Twenty full-time international University students (60% female, 90% aged 18–25 years and 65% undergraduates) were recruited. Students reported (i) stress from personal (e.g., worries about health and academic attainment), interpersonal (e.g., perceived prejudice and lack of social support), and environmental factors (e.g., uncertainties about academic programme and unclear COVID-19-related information); (ii) significant differences in culture and cognitive appraisal in the levels of perceived susceptibility and severity; (iii) positive thinking and using alternative measures in meeting challenges, which included effective emotion and problem coping strategies, and the importance of support from family, friends and schools; and (iv) negative psychological impact (e.g., worries and stress) and positive personal growth in crisis management and gains in family relationships.

**Conclusions:** With the rise in sinophobia and uncertain developments of the pandemic, proactive support from government and academic institutions are urgently needed to reduce stress and promote the well-being of international students, especially Chinese students in the U.K. and U.S. Clear information, public education and policies related to the pandemic, appropriate academic arrangements from universities and strong support systems play important roles in maintaining students' psychological health.

**Clinical Trial Registration:** The study was registered with the National Institutes of Health (https://clinicaltrials.gov/, identifier: NCT04365361).

## Introduction

The COVID-19 pandemic has caused widespread psychological distress, likely leading to a long-term upsurge in the incidence and severity of mental health problems (1). With most of the resources and measures rightly prioritized on infected patients, frontline health workers and related clinical research, more attention should also be placed on students, as the pandemic has caused significant disruptions to education and learning. Students reported concerns regarding their studies and future professional careers, and experienced boredom, anxiety, and frustration (2).

University students adapting to new academic environments and demands often face novel challenges that adversely affect their mental well-being (3). International students endure additional stressors such as living abroad alone and adjusting to the host country's culture and norms (4). During the COVID-19 pandemic, Asian communities in Western countries, such as in the United Kingdom (U.K.) and United States (U.S.), have been targets of incidents and attacks involving xenophobia, racism and discrimination (5). Violent, unprovoked attacks against Asians in both the U.K. and U.S. began to spike in February 2021, prompting widespread condemnation from global leaders and collective acts of support and solidarity from different communities (6).

Hong Kong has had previous experiences with mass masking for the control of the severe acute respiratory syndrome (SARS) epidemic in 2003. Majority of students from Hong Kong had therefore learned that facemask wearing, social distancing and seeking timely medical advice can prevent the spread of disease. Hong Kong had almost 100% voluntary mass masking in public places very quickly since the start of COVID-19 in early February 2020, when the outbreak was rapidly under control with no lockdown (7). However, epidemic prevention guidelines from different agencies and organizations, including the World Health Organization and U.K. and U.S. governments, conflicted with practices and experiences in students' home cities. Distrust in the government's preventive measures and guidelines were also observed in students studying in the U.K. (8) and U.S. (9). Furthermore, the potential of information overload (both correct and incorrect information) and being away from central social support systems such as family and friends during the pandemic can be greatly challenging (10). International students may have felt more isolated compared to local students due to different cultural, economic or language barriers and additional stress from lockdowns, closed borders, and difficulties securing air tickets to fly home which was perceived as (and was in fact) safer.

The Transactional Model of Stress and Coping Theory is a cognitive framework that emphasizes the evaluation of threat (stressors), challenges (cognitive appraisals), coping (strategies and support systems) and harms (negative psychological impacts) (11). This theory has been widely used in various fields, including the process of coping with work stress (12), psychological adjustment to cancer (13), and as a tool for understanding retention in HIV care (14). Our published sister paper used the components of this model to examine students' stressors, coping strategies and mental health impacts *via* an online questionnaire survey (15).

We searched PubMed on 12 November 2021 using keywords including “international students” and “COVID-19,” and found 15 articles exploring the stress and mental health of international students studying in China (16–20), South Korea (21), U.K. (15, 22), U.S. (15, 23), Australia (24, 25), Russia (26), Turkey (27), and Poland (28, 29). Six studies were on Chinese or Asian international students (15, 21–23, 25, 29) and nine studies focused on students of other ethnicities (16–20, 24, 26–28, 30). We found no qualitative articles that systematically investigated COVID-19-related stressors, coping and positive and negative impacts in international students during the pandemic.

The current study aimed to obtain in-depth information to explore the positive and negative experiences and impacts from COVID-19 of a group of Hong Kong Chinese international students studying in the U.K. and U.S. during the pandemic. Specifically, the research questions were: (i) What were the experiences of Chinese international students studying in the U.K. and U.S. and the factors influencing their stress (stressors) amidst the pandemic?; (ii) How did students perceive their susceptibility and severity in relation to the pandemic (cognitive appraisals)?; (iii) What were their coping strategies to handle the challenges from the pandemic?; (iv) Who were their support systems to help them face difficulties, and (v) What were the impacts on psychological health, personal growth and family relationships?

## Methods

### Study Design

We used a descriptive phenomenological approach to understand Chinese international students' experiences and perceptions in the early stage (Mar to May 2020) of the COVID-19 pandemic. This approach is a qualitative methodology that aims to capture the lived experiences of individuals and describe the meanings of such experiences from an insider perspective (31). The period studied was the time students were studying in the U.K. or U.S., and during the quarantine period in H.K. after they returned, if applicable. Purposive sampling was used to recruit those students who joined the online survey (15). Online focus group interviews were conducted instead of face-to-face gatherings to comply with social distancing recommendations and regulations.

### Characteristics of Lead Researcher

The lead researcher (AL) is a female University academic, behavioral scientist and registered nurse with two doctoral degrees in nursing and public health, and more than 25 years of clinical nursing, teaching and research experience. She was responsible for asking the semi-structured theory-based questions, which was in line with the Transactional Model of Stress and Coping Theory. A research assistant with a master's degree in psychology was responsible for taking notes during the interviews to record important points from the interviewees. Another research assistant with a master's degree in sociology was responsible for monitoring participants' responses and ensuring active participation. All pre-set questions were discussed to ensure all components of the theory were included.

### Sampling Strategy

The participants were recruited through a cohort that joined a questionnaire survey, in which details have been described in our sister paper (15). We first encouraged students who completed the online survey to provide their phone numbers for further contact. We then invited the students *via* WhatsApp messages and phone calls to join the online focus group interviews. We recruited both female and male students with different years and fields of study, programme and study country to allow maximum variation sampling. The inclusion criteria targeted full-time international University students aged 18 years or older studying abroad in the U.K. or U.S. All individuals voluntarily participated in the study.

### Ethical Issues Pertaining to Human Subjects

Ethics approval was granted by the Institutional Review Board of The University of Hong Kong / Hospital Authority Hong Kong West Cluster (reference number: UW20-298). A link to a form with the study objectives and informed consent was sent to each participant who consented electronically before the online interviews were conducted. The voluntary nature of the study was explained to students. Confidentiality was assured by using numbers instead of names and removing identifying information from the transcripts. All audio recordings and transcripts were saved on a password-protected computer.

### Data Collection Methods

Four 1.5-h online semi-structured interviews were conducted from 3 to 12 May 2020 *via* Zoom, a cloud-based video conferencing service. An interview link was sent to each participant *via* WhatsApp. Interviews were audio-recorded. Before starting the interviews, interviewees answered a brief questionnaire about their study background. The researchers remained neutral in data collection and built a good rapport with interviewees. Unconditional acceptance, active listening and clarifications were adopted during data collection to enhance data authenticity and avoid bias.

### Interview Outline

**Figure 1:**
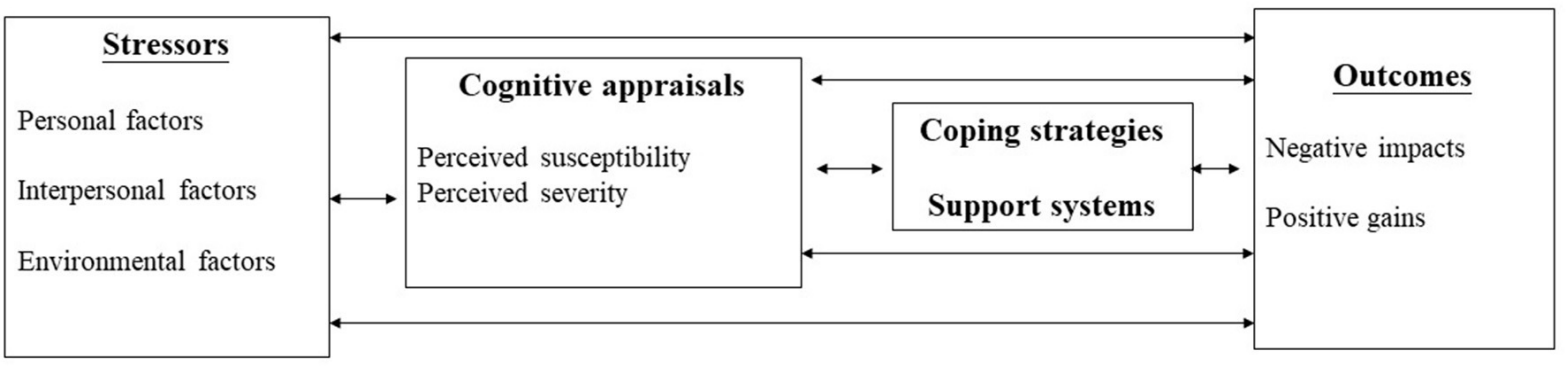
The four components from Transactional Model of Stress and Coping Theory.

A theory-based semi-structured interview guide was modified from the components of the Transactional Model of Stress and Coping Theory (32). A framework approach was used to guide the process of thematic analysis (33). Figure 1 shows the four components, including stressors, cognitive appraisals, coping strategies and support systems, and outcomes (negative impacts and positive gains) related to COVID-19. Questions were structured chronologically to aid recall. The sample questions asked during the interviews are summarized in Table 1.

**Table 1:** Sample questions for the theory-based interviews.

**Stressors**	
Trigger question: How did you generally feel whilst you were in your country of study?	
Personal factors	How do you personally feel about the pandemic?
	What factors affected your decision to return or not return to HK?
Interpersonal factors	Did you ever hear about, observe, or experience prejudice or discrimination related to COVID-19?
	Why do you think people may have acted in this way?
	How did hearing about, observing, or experiencing these things make you feel?
Environmental factors	How do you arrange your study plan and placement (if any)?
	Where do you get information about the outbreak?
	Do you feel like you could have done better academically if more support or information was given to you by your school?
**Cognitive appraisals**	
Trigger question: What was your perception of COVID-19 during January 2020 to March 2020 when you were in your country of study?	
Perceived severity	How severe did you believe the outbreak was?
Perceived susceptibility	How susceptible to / at risk of infection did you feel you were?
	Did you feel in control of your own situation?
**Coping effort and support systems**	
Trigger question: How did you cope with the negative emotion or events, if any, during the pandemic?	
Emotion regulation	What do you do to lighten up your mood?
	Were you able to control your own emotions?
	Can you give me an example of how you did this?
Problem management	How did you solve problems whilst alone overseas and not with friends or family?
Trigger question: What support has been the most valuable to you throughout the outbreak?	
Family support	Tell me about your quarantine experience at home.
	What was your interaction with your family members like during the 14-day period immediately after returning to HK?
Peer support	Tell me about what your preparations and journey home was like? Did you and your friends mutually support each other during the pandemic and how?
School support	How do you feel about the arrangement or support that you received from your school?
**Outcomes**	
Trigger question: Would you say that there were any experiences that influenced your personal growth and resulted in any positive or negative impacts?	

### Data Processing

The interview recordings were transcribed verbatim and analyzed using directed content analysis. As analyses proceeded, additional codes were developed, and the initial coding scheme was revised and refined.

### Data Analysis

Two researchers (AL and AC) independently reviewed the interview materials (including transcripts and recordings), summarized, and extracted meaningful statements. Field notes were reviewed during the analysis process. The software NVivo 11.0 (QSR International; Melbourne, VIC, Australia) was used to assist qualitative data administration, including creating codes, organizing, and summarizing data, searching for interrelationships between codes, and suggesting themes. We used a mixed-methods triangulation design to corroborate the findings between qualitative and quantitative data from our sister paper (15). To ensure the quality of this study and paper, we followed the Standard for Reporting Qualitative Research in implementation and reporting (34).

### Techniques to Enhance Trustworthiness

Different strategies were used to enhance the trustworthiness of the research findings, including credibility (the truthfulness of data), dependability (the stability of data), confirmability (the congruence of data), and transferability (the applicability of data). To enhance study credibility, member checking was conducted by asking participants (one student from each focus group interview) to review the transcripts from interviews they participated in and give feedback on emerging interpretations to ensure a good representation of their realities. Each interview was analyzed by two researchers. Peer debriefing was then held to review the consistency of identified information with other co-investigators. We also triangulated the qualitative findings with the quantitative results from our previously published sister paper (15) and secondary data (e.g., information from newspapers and multi-media sources) to ensure the truthfulness of the findings. To enhance study dependability, the description of the coding and the descriptions of themes were checked and reconfirmed by a researcher who was not involved in data collection (SS). To promote study confirmability, an audit trail was conducted by making field notes when conducting interviews to allow tracing of the course of research. Most importantly, we clearly reported the details of study design, characteristics of investigators, participants, sampling strategies, data collection and analysis procedures to promote study transferability.

## Results

### Participants

Twenty-five students who joined the online survey outlined in our sister paper (15) provided their phone numbers and were contacted and invited to join the four focus group interviews. Five students refused. Table 2 shows the characteristics of 20 students (60% female, 90% aged 18–25 years, 65% undergraduates, 15% first-year students, 30% final year students, 40% studying programmes with a placement component, 40% studying medical and health-related programmes, 75% returned to hometown) who joined the zoom focus group interviews conducted on 3 May, 8 May (2 sessions), and 12 May 2020.

**Table 2:** Students' characteristics.

**No**.	**Age**	**Sex**	**Country of education**	**Education programme level**	**First year**	**Final year**	**Field of study**	**Placement**	Returnee/Stayer^*^
1	20	Male	U.K.	Bachelor	No	No	Aviation Engineering	No	Returnee
2	18	Female	U.S.	Bachelor	Yes	No	Music	No	Returnee
3	25	Male	U.K.	Master	No	Yes	Biomedical Engineering	No	Stayer
4	20	Male	U.K.	Bachelor	Yes	No	Sports and Health Science	No	Returnee
5	22	Female	U.K.	Master	No	No	Chiropractic	Yes	Returnee
6	21	Male	U.K.	Bachelor	No	Yes	Engineering	No	Returnee
7	22	Male	U.K.	Master	No	No	Chiropractic	Yes	Returnee
8	21	Male	U.K.	Bachelor	No	Yes	Economics	No	Returnee
9	22	Female	U.K.	Master	No	No	Chiropractic	Yes	Returnee
10	23	Male	U.K.	Master	No	Yes	Public Policy	No	Stayer
11	21	Female	U.K.	Bachelor	No	Yes	Business	No	Returnee
12	23	Male	U.K.	Bachelor	No	No	Physiotherapy	Yes	Returnee
13	20	Female	U.K.	Bachelor	No	No	Chemistry	No	Returnee
14	20	Female	U.K.	Bachelor	No	No	Chemistry	No	Returnee
15	19	Female	U.K.	Bachelor	Yes	No	Pathology	No	Returnee
16	26	Male	U.S.	Master	No	No	Theology	No	Stayer
17	26	Male	U.S.	Master	No	No	Theology	No	Stayer
18	20	Female	U.K.	Bachelor	No	Yes	Physiotherapy	Yes	Stayer
19	20	Female	U.K.	Bachelor	No	No	Physiotherapy	Yes	Returnee
20	21	Female	U.S.	Bachelor	No	No	Business	No	Returnee

* *As of the participant's focus group discussion date*.

*Returnee, Returned to hometown; Stayer, Stayed at institution country*.

### COVID-19-Related Stressors

Students reported stress from various sources, including individual, interpersonal, and environmental stressors, which adversely affected their psychological health. Worries about personal health and difficulty locating personal protective equipment were the most common individual stressors. Large discrepancies between students' practices and experiences in their hometown and country of study in relation to COVID-19 prevention and management and unclear arrangements from universities also caused additional stress regarding academic attainment.

“*At the start of the pandemic, I was so stressed because it was really difficult to locate face masks and other protective gear… we knew we had to protect ourselves*” (*Participant 7, male, studying in the U.K*.).

“*The government repeatedly told us not to wear facemasks and not to worry… this made me scared since I know wearing one is an effective preventive method… We flew back to Hong Kong as we felt that it would be a safer place for us to stay*” (*Participant 19, female, studying in the U.K*.).

“*I felt that my school was really unprepared, and the arrangements were not so good… made me really frustrated because they didn't announce any plans for examinations, how tests will be scored… This would have a big impact, especially for those of us in our final year. I understand schools have a lot to do, but nothing was clear*” (*Participant 15, female, studying in the U.K*.).

Prejudiced attitudes and behaviors of others (such as toward facemask wearing) and the lack of social support (such as unavailability of air tickets to fly back home) were the major interpersonal stressors.

“*In February, while my friends and I were walking down the street wearing facemasks, someone yelled* ‘*coronavirus!*’ *from across the street, labeling us as the virus*” (*Participant 15, female, studying in the U.K*.).

“*I understand that they (Westerners) feared the virus…but Sinophobia is real. I don't want to wear face masks in the U.K. as I do not want others to label me as a Chinese person from China*” (*Participant 10, male, studying in the U.K*.).

“*We are unable to book a flight to Hong Kong at an acceptable price, and there were so many things to settle within a short period*” (*Participant 19, female, studying in the U.K*.).

Uncertainties about their academic programme, unclear COVID-19-related information and ineffective local outbreak management were the commonly reported environmental stressors.

“*My school suspended classes for nearly a month after face-to-face classes were canceled. They didn't upload revision materials or notes to the portal until a week before examinations… There was too much uncertainty, and I had no clue what was going to happen next*” (*Participant 1, male, studying in the U.K*.).

“*I do not dare to leave the U.K.… what if my school suddenly changes their course or examination arrangements? There is too much uncertainty*” (*Participant 10, male, studying in the U.K*.).

“*I felt like the U.K. government was too laidback at the beginning of the pandemic and had no focus… we weren't given much information… they only mentioned that it was not necessary to wear face masks, and to just practice social distancing… but London is densely populated like Hong Kong. Social distancing alone is not useful*” (*Participant 3, male, studying in the U.K*.).

### Cognitive Appraisals

Although students understood the severity of the outbreak situation, those around them did not feel the same danger or urgency. Students felt the cultural differences in the level of perceived susceptibility and severity were significant.

“*During my internship, my supervisors comforted patients by telling them there was nothing to worry about with the virus. I guess they didn't want to cause panic… But I felt this gave patients the wrong impression about the severity of the virus*” (*Participant 12, male, studying in the U.K*.).

“*As health care professional students, we were conscious and aware about the outbreaks. We were shocked that our teachers had no idea about how serious the situation was and the infectious nature of the virus when we mentioned it to them. We explained our concerns and referenced the SARS outbreak, but they didn't really care and felt like we were exaggerating*” (*Participant 9, female, studying in the U.K*.).

“*I felt like we were in a very worrying situation, as our friends, schools, and even the government did not understand the seriousness of the situation…we could possibly die from COVID-19 if we got infected!*” (*Participant 2, female, studying in the U.S*.).

### Coping Strategies and Support Systems

Students adopted two main types of coping methods: emotion regulation and problem management. Emotion regulation included talking with family and friends to ease loneliness, connecting with friends going through the same situation to feel more understood, and practicing positive thinking. For problem management, students took a proactive step of using alternative measures to protect themselves in meeting challenges during the pandemic. Family, peers, and schools were the three main pillars of support for students and helped provide psychological support and relief from the challenges brought on by the pandemic.

“*While quarantining at home, I facetimed with my parents everyday even though we were living together under the same roof. They would check in on me and bring me things to ease my loneliness and make me feel better. We would have dinner together every night via FaceTime… Chatting with them made me feel better*” (*Participant 9, female, studying in the U.K*.).

“*I can't control what other people say. Thus, I choose not to take what they say to heart. I just focused on what happened to me positively*” (*Participant 1, male, studying in the U.K*.).

“*I'm proud of myself for being resourceful… when I couldn't buy any face masks, I used my scarf and turned it into a face covering*” (*Participant 16, male, studying in the U.S*.).

### Negative Impacts and Positive Gains

Students also reported feeling worried and stressed in the early stage of the pandemic.

“*I had to explain to my teachers why I was worried, especially with my experience from SARS…*” (*Participant 5, female, studying in the U.K*.).

“*I was so stressed because it was really difficult to locate face masks and other protective gear*” (*Participant 7, male, studying in the U.K*.).

However, they also reported personal growth and enhanced family communication and relationships.

“*From this experience, I felt very smart to be able to handle so many things in a short period… confirming my flights, canceling rental agreements, and shipping my belongings to a warehouse… I sensed and appreciated my improvements in time management and communication skills*” (*Participant 13, female, studying in the U.K*.).

“*While quarantining at home, I facetimed with my parents every day even though we were living together under the same roof. They would check in on me and bring me things to ease my loneliness and make me feel better. We would have dinner together every night via FaceTime… chatting with them made me feel really good and closely connected*” (*Participant 8, male, studying in the U.K*.).

“*I never knew my emotional attachment to my parents was that deep… until now. I missed them so much and was worried about their health when we were apart*” (*Participant 18, female, studying in the U.K*.).

## Discussion

This is the first online focus group study on the experiences of Chinese international University students studying in universities in the U.K. and U.S. during the pandemic. Students reported their stress, perceived susceptibility and severity, coping strategies of positive thinking, the importance of support from family, friends and school, and positive gains in personal growth and family relationships.

### Experiences of Students and Factors of Stress

The COVID-19 pandemic has caused unprecedented disruptions to learning and placed enormous strain on University students in a transitional and challenging period of life. Our findings showed that most students lacked confidence in completing their programs on time with satisfactory grades, especially those with placement components (35). A UK study showed school closures and cancellation of placement during COVID-19 led to learning loss. Students made little or no progress while learning from home, and authors suggested losses were even larger in countries with weaker infrastructure or longer school closures (36). Additionally, the high level of stress among international students could be explained by cultural differences; in most Asian cultures, success is viewed through academic performance (37), with a strict emphasis on educational effort and attainment as a source of pride, and Asian students having more stringent work ethics and higher educational aspirations.

Another critical issue highlighted in our findings is the racial stereotypes and discrimination against Chinese international students. Students shared their experiences regarding verbal confrontations and shunning behaviors in local communities. They were being portrayed as the vector of the virus and accused by locals as the cause of the pandemic. The issue of discrimination and stigmatization against those of Chinese or East Asian descent would also be detrimental to the well-being of Chinese international students. Indeed, the uprising of racial prejudice toward people of Chinese or East Asian descent throughout the pandemic has been frequently reported in the literature (38–40). Racist stigmatization of ethnic and racial minorities are identified as a strong predictor of early onset of mental and psychiatric disorders in adolescents and young adults that significantly associated with elevated incidences of feelings of isolation, depression, substance abuse, and self-inflicting behaviors (29, 41), and affect career development (42). Given the profound impacts of discrimination and stigmatization against Chinese and East Asian students on their well-being, immediate actions should be taken to tackle such xenophobia. Researchers suggested that racial prejudice and biased media were the primary sources fuelling sinophobia in the West, and thus regulations should be considered in preventing such problems and promoting social inclusion (43).

Moreover, students reported in the interviews that information regarding the pandemic situation was chaotic and confusing. They suggested that governments of their host countries did not provide consistent and standardized information (such as precautions, diagnosis and treatment, and related policies) to students and the public, particularly in the early stage of the pandemic (44). Social media platforms also played an essential role in rapidly delivering COVID-19-related information during the pandemic. However, due to a lack of a standardized and trustworthy source of information on these platforms, the spread of misleading and confusing information has been noted, which was referred to as an “infodemic” (45). For instance, myths and conflicting information regarding the need to use facemasks to reduce the likelihood of infection at the beginning of the pandemic created confusion in the use and selection of facemasks in the general public (46). This “infodemic” was also associated with panic buying behaviors in the community, resulting in the general public stocking up of resources such as facemasks and disinfectants in the first few months of the outbreak (47), and the reduction in vaccination intention among the general public due to inconsistent information on the internet regarding the effectiveness of the vaccine, even though it is considered the most effective measure in controlling the pandemic (48).

In the present findings, we found discrepancies regarding the risk perceptions toward the pandemic (e.g., the severity of the pandemic and the susceptibility of infection) between people from East Asia and the West in the early pandemic stage. Students from mainland China and Hong Kong had a generally strong awareness of infection control measures and infectious disease management because of their experiences from previous pandemics. A study conducted in Hong Kong indicated that concerns regarding the COVID-19 pandemic were greater in individuals who experienced the previous severe acute respiratory syndrome (SARS) epidemic in 2003 than those who had no such prior experience (49). These previous experiences had led to a higher capability and willingness to implement infection control and disease management measures in dealing with the existing COVID-19 pandemic. This might explain the high-risk perception and alertness among the Chinese international students in our study.

### Coping Strategies and Support Systems

Under various stressors, our students tended to seek support from their peers and significant others to address unpleasant feelings pertaining to the situation, such as loneliness and boredom due to quarantine and lockdown. Interestingly, although the importance of social support has been frequently reported as a primary resource for younger people in emotional regulation and support, it has been reported as the least preferred coping strategy among University students facing COVID-19-related challenges. For example, a study in Pakistan showed that University students preferred to deal with problematic issues on their own (50). These results could be related to the isolation and social distancing during the pandemic, causing difficulties for students to maintain contact with their family and friends and obtain social support. For international students, they have adapted to maintaining contact with their parents and friends despite the geographical constraints, which might promote their reliance on social support during the pandemic. Although our study focused on international but not local University students, the results might offer insights into maintaining social support for local students under isolation during the outbreak period. Strategies in promoting social interactions of local students with their friends and family through virtual platforms, such as teleconferencing, could be suggested and promoted by schools and universities under the current arrangements of quarantine and social distancing measures.

Additionally, our students adopted two main coping strategies: emotion-focused and problem-focused coping. For emotion-focused coping, students practiced positive thinking in the face of adversity, showing acceptance of and displaying assertive attitudes toward the current situation. For problem-focused coping, students took proactive steps to identify alternative measures in dealing with challenges, such as supporting each other by sharing resources. These behaviors highlighted their adaptability and ability to adjust to new situations by displaying their capacity in both an assertive and proactive manner, which have been reported in other studies that highlighted the coping strategies of University students amid adversity (50, 51). However, some students might adopt maladaptive coping strategies in the face of the stressors associated with the pandemic, such as interpersonal withdrawal and avoidant behaviors (52), which can adversely affect their mental and psychological capacity in handling challenging situations, and lead to adjustment problems that could further cause depressive mood, anxiety, or misconduct behaviors (53). Thus, schools and universities play an important role in helping students adjust to such an unprecedented situation by addressing their needs and identifying those who require further help and follow-up.

### Positive Gains on Personal Growth and Family Relationships

In the face of adversity, most of our students stated that they had experienced positive personal growth throughout the process of coping with challenges during COVID-19. Although the students experienced a certain level of emotional distress, they demonstrated psychological resilience to bounce back from adversity and foster personal growth in terms of self-efficacy and family cohesion. Their capacity in coping and resiliency demonstrated their growth and grit throughout the pandemic. The growth mindset places emphasis on personal improvement and refinement through experiencing or learning to handle and overcome challenges, while the construct of grit refers to the perseverance of individuals to persist during adversity or after setbacks (54). These attributes are essential traits in predicting emotional distress and psychological resilience in University students during adversity. In the literature, the level of resilience and adoption of adaptive coping strategies and social support are considered the primary mediators of emotional distress in students in China during the COVID-19 outbreak (55). Consistent with the findings of the present study, it is suggested that a stressful event managed in an adaptive manner can induce positive growth among the individuals who have experienced the situation (56). Such growth has also been described as post-traumatic growth related to exposure to traumatic experiences (57). Post-traumatic growth has been identified as a protective factor preventing individuals from developing post-traumatic stress disorder after a major life event (58). Therefore, it is important for governments and academic institutions to develop students' resilience to expand their psychological capabilities in addressing unprecedented challenges, which can help protect them from being overwhelmed during the current pandemic and assist them to achieve personal growth amidst various hardships.

### Limitations

Our study had several limitations. First, this study was limited by snowball sampling. Although this strategy was effective to recruit suitable respondents efficiently in a short period of time, we might have recruited students with similar characteristics or traits. Second, as the experiences of students may be unique due to their background and the relatively small sample size, the results might have limited generalizability of the findings. However, we triangulated the findings with our previous research (15, 22) and secondary data from social media and other publications regarding the experiences of international students (16–19, 23, 26, 30, 43), which would support reasonable generalisability. Further studies with large sample size and international students from other countries are needed. Third, this study was conducted in May 2020, and students were invited to recall their time studying overseas, and recall bias was possible. However, the recall was the only feasible way to obtain students' information on their experiences. Lastly, as the interviews were held *via* an online video conference platform, and although all respondents were willing to turn on their cameras, observing their body language was difficult. However, the use of an online platform was the most appropriate method under social distancing regulations.

### Implications

During the COVID-19 pandemic, Chinese international students were facing different challenges and enduring various stressors that required support. This paper is historical evidence on the experiences and needs of Chinese international University students studying in the U.K. and U.S. during the pandemic. With more international students gradually returning to their respective schools amidst the ongoing pandemic and rising xenophobia, governments, and academic institutions should take immediate and proactive action to provide additional support and promote students' mental well-being. At the individual level, students' resilience should be enhanced to recover quickly from challenging situations, for example, learning specific strategies in stress management such as positive thinking, breathing exercises, and communicating their concerns and worries (59). More studies on the nature, role, and impact of resilience, in particular the coping methods to relieve students' stress, and the pros and cons of online and face-to-face interviews are warranted. At the University level, prompt actions taken by teachers and schools, such as reduced course loads and flexible grading systems, can help alleviate stress among students. Universities should devise strategies to protect international students from harmful misconceptions and enhance communication among students of different cultural or ethnic backgrounds to facilitate mutual understanding between local and international students (29). At the government and policymaking level, clear information and public education related to the pandemic should be communicated. Preventing such behaviors should also be enforced by national authorities (29). Professional organizations should host cross-sectoral meetings such as webinars or forums where different stakeholders can share their experiences and make suggestions on appropriate arrangements from universities and related parties. Strong support systems also play important roles in maintaining students' psychological health.

## Conclusions

Chinese international students described their stressors and coping strategies during the COVID-19 pandemic, resulting in both adverse psychological impacts and positive personal growth and family relationships. With the rise in sinophobia and uncertain developments of the pandemic, proactive support from government and academic institutions are urgently needed to reduce stress and promote the well-being of international students, especially Chinese students.

## Data Availability Statement

The datasets in the article are not readily available because the sharing of data to their parties was not mentioned in subject's consent. Requests to access the datasets should be directly contact corresponding author.

## Ethics Statement

Ethics approval was granted by the Institutional Review Board of The University of Hong Kong/Hospital Authority Hong Kong West Cluster (reference number: UW20-298). The patients/participants provided their written informed consent to participate in this study.

## Author Contributions

AY-KL led the conception and design of the survey. AY-KL, AC-MC, and DY-SY carried out the survey. AY-KL, AC-MC, SM-MS, SK-KL, and T-HL were responsible for interpreting the data and drafting the manuscript. TT-KL, MS-MI, and T-HL were closely involved in data interpretation and manuscript revision. All authors read and approved the final manuscript.

## Funding

This study was funded by Sir Robert Kotewall Professorship in Public Health Fund.

## Conflict of Interest

The authors declare that the research was conducted in the absence of any commercial or financial relationships that could be construed as a potential conflict of interest.

## Publisher's Note

All claims expressed in this article are solely those of the authors and do not necessarily represent those of their affiliated organizations, or those of the publisher, the editors and the reviewers. Any product that may be evaluated in this article, or claim that may be made by its manufacturer, is not guaranteed or endorsed by the publisher.
